# The three-dimensional displacement tendency of teeth depending on incisor torque compensation with clear aligners of different thicknesses in cases of extraction: a finite element study

**DOI:** 10.1186/s12903-022-02521-7

**Published:** 2022-11-16

**Authors:** Yuxun Cheng, Xulin Liu, Xin Chen, Xin Li, Shishu Fang, Wei Wang, Yanning Ma, Zuolin Jin

**Affiliations:** 1 State Key Laboratory of Military Stomatology & National Clinical Research Center for Oral Diseases & Shaanxi Clinical Research Center for Oral Diseases, Department of Orthodontics, School of Stomatology, Air Force Medical University, Xi’an, 710032 China; 2 Urumql DW Innovation InfoTech Co. Ltd, Xinjiang, 830000 China; 3 Shanxi Medical University School and Hospital of Stomatology, Taiyuan, 030001 China

**Keywords:** Finite element analysis, Clear aligner, Torque, Aligner thickness, Power ridges

## Abstract

**Background:**

Despite the popularity of clear aligner treatment, the effect of the thickness of these aligners has not been fully investigated. The objective of this study was to assess the effects of incisor torque compensation with different thicknesses of clear aligner on the three-dimensional displacement tendency of teeth in cases of extraction.

**Methods:**

Three-dimensional finite element models of the maxillary dentition with extracted first premolars, maxilla, periodontal ligaments, attachments, and aligners were constructed and subject to Finite Element Analysis (FEA). Two groups of models were created: (1) with 0.75 mm-thick aligners and (2) with 0.5 mm-thick aligners. A loading method was developed to simulate the action of clear aligners for the *en masse* retraction of the incisors. Power ridges of different heights were applied to both groups to mimic torque control, and the power ridges favoring the translation of the central incisors were selected. Then, we used ANSYS software to analyze the initial displacement of teeth and the principle stress on the PDL.

**Results:**

Distal tipping, lingual tipping and extrusion of the incisors, distal tipping and extrusion of the canines, and mesial tipping and intrusion of the posterior teeth were all generated by clear aligner therapy. With the 0.5 mm-thick aligner, a power ridge of 0.7 mm could cause bodily retraction of the central incisors. With the 0.75 mm-thick aligner, a power ridge of 0.25 mm could cause translation of the central incisors. Aligner torque compensation created by the power ridges generated palatal root torque and intrusion of the incisors, intrusion of the canines, mesial tipping and the intrusion of the second premolar; these effects were more significant with a 0.75 mm-thick aligner. After torque compensation, the stress placed on the periodontal ligament of the incisors was distributed more evenly with the 0.75 mm-thick aligner.

**Conclusions:**

The torque compensation caused by power ridges can achieve incisor intrusion and palatal root torque. Appropriate torque compensation with thicker aligners should be designed to ensure bodily retraction of anterior teeth and minimize root resorption, although more attention should be paid to the anchorage control of posterior teeth in cases of extraction.

## Background

Since the 1990s, clear aligner therapy (CAT) has developed rapidly. In contrast to conventional fixed treatment, CAT involves a form of tooth movement that features a predetermined “mismatch” between the aligner and the tooth [1]. CAT provides adult patients with a significant range of aesthetic treatments that do not negatively affect their social lives or relationships [2–4] and are associated with fewer periodontal complications and a lower risk of root resorption [5, 6]. It is generally agreed that aligners are particularly efficient at resolving malocclusion of slight to moderate complexity in non-extraction cases due to their good capacity to expand, align and level the arches [7]. However, no clear clinical recommendations, based on solid scientific evidence, have been made for complex cases such as cases of extraction [7, 8].

Finite Element Analysis (FEA) refers to the use of a mathematical dentition model derived from by computer aided design (CAD) and estimates the stresses generated within different tissues, such as alveolar bone, the PDL and teeth; FEA can also determine the loading and displacement patterns of all structures. This form of analysis can contribute to our biomechanical understanding of orthodontic devices. Over recent years, FEA has been widely applied in different fields of dentistry, from fixtures to the simulation of dental movements [9–14]. An increasing body of evidence demonstrates that researchers are applying this technology to investigate the biomechanical principles underlying the use of clear aligners.

Researchers have conducted several studies on the biomechanics of cases of extraction with a clear aligner from many different perspectives using FEA. Gomez et al. established a finite element model (FEM) in a canine model to simulate bodily movement and clarified that composite attachments generate a force system that can produce bodily tooth movement [15]. In another study, Liu constructed a FEM for maxillary dentition and demonstrated that appropriate overtreatment, by placing attachments on the canines, should be designed to ensure the bodily retraction of anterior teeth in cases of extraction [16]. Our previous work showed that the required torque control for upper anterior teeth with oversize axial inclination was weaker than that for upper anterior teeth with normal axial inclination [17]. Although many studies have analyzed the biomechanics of aligner-attachments and torque control of anterior teeth, the thickness of aligner has not been investigated in full detail, even though this factor is closely associated with other important features such as the forces and moments they generate and the displacement tendency of teeth [18, 19]. Elshazly and Elkholy demonstrated that orthodontic forces increased as aligner thickness increased, thus proving that there was a significant influence of the aligner thickness on their biomechanical functionality [20, 21]. Relevant studies previously focused on aligner thicknesses ranging from 0.5 mm to 0.75 mm [21–23]. Therefore, we analyzed the relative influence of the 0.75 mm-thick aligner and the 0.5 mm-thick aligner, with incisor torque compensation, on the three-dimensional displacement tendency of teeth in cases of extraction.

In this study, we conducted FEA and evaluated the effects of incisor torque compensation with different thicknesses of clear aligner on the three-dimensional displacement tendency of teeth in cases of extraction. Our overall goal was to provide direction for the use of clear aligners in the clinic.

## Methods

### Main materials and equipment

Cone beam computed tomography (CBCT) data (HiRes3D-Plus; Largev, Beijing, China); MIMICS 20.0 software (Materialise, Leuven, Belgium); GEOMAGIC Studio 2014 (RaindropGEOMAGIC, North Carolina, USA); NX1911 software (Siemens, German); ANSYS Workbench 2019 (ANSYS, Pennsylvania, USA); clear aligner (Align Technology, San Jose, California, USA); attachment (Filtek™P60, 3 M ESPE, 3 M, St. Paul, MN, USA).

### Relevant statement

The ethics committee of School of Stomatology, Air Force Medical University, China, approved the study (IRB-REV-2022059). The authors confirmed that all the methods were performed in accordance with the relevant guidelines and regulations.

### Construction of the model

**Fig. 1:**
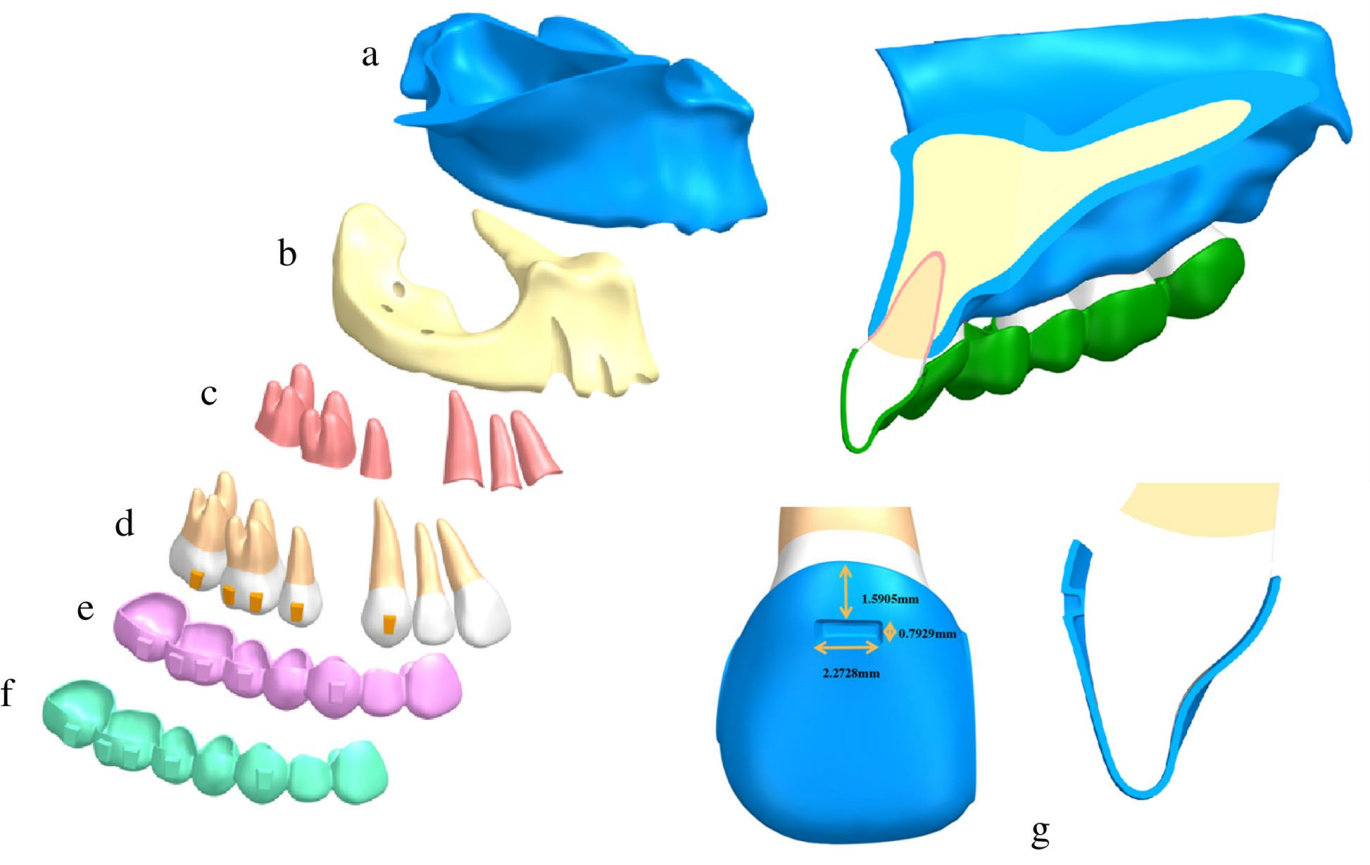
The structure of the finite element model **a** bone cortex; **b** bone cancellous; **c** periodontal ligament; **d** teeth; **e** 0.5 mm-thick aligner; **f** 0.75 mm-thick aligner; **g** the location and dimension of power ridge

CBCT data (HiRes3D-Plus; Largev, Beijing, China) were acquired from a healthy volunteer with well-aligned dentition and a healthy periodontal condition. First, the data were imported into MIMICS 20.0 software (Materialise, Leuven, Belgium) to generate three dimensional base models of the maxillary dentition with the extracted first premolar. Then, we used GEOMAGIC Studio 2014 (RaindropGEOMAGIC, North Carolina, USA) to optimize the basic model and create a surface model structure. With the help of NX1911 software (Siemens, German), the outer surface of the maxillary teeth roots was extended outwards by 0.25 mm to generate a preliminary model for PDL. The maxillary was also moved inwards by 1.3 mm to generate cortex bone and cancellous bone structures. Vertical rectangular attachments (3 mm height, 2 mm width, and 1 mm thickness) were designed for all teeth other than the central incisor and the lateral incisor. The maxillary crown and attachment were extended outwards by 0.5 mm/0.75 mm to simulate the thickness of the appliance (the clear aligner was designed according to Clincheck software of the Invisalign system) and each tooth was treated as an independent component (Fig. 1). Finally, all components were imported into ANSYS Workbench 2019 (ANSYS, Pennsylvania, USA) to generate a three-dimensional finite element model for FEA.

Model meshing was constructed with 10-node tetrahedral elements. Tetrahedral elements were connected to each other by nodes for the transmission of stress function. The number of nodes and elements for each component of the model are shown in Table 1. Significantly, compared with the 0.5 mm-thick aligner, the number of nodes for 0.75 mm-thick aligner increased by 733, and the number of elements for 0.75 mm-thick aligner increased by 491. The element size for alveolar bone was 2.0 mm; for the PDL and attachments, the elemental size was 0.5 mm; for teeth and the aligner, the elemental size was 1.0 mm.

**Table 1:** Number of Nodes and Elements of the Components of the FEM

Material	Number of elements		Number of nodes	
	0.5 mm-thick aligner	0.75 mm-thick aligner	0.5 mm-thick aligner	0.75 mm-thick aligner
Attachment	2291		4532	
Tooth	40,614		59,335	
PDL	39,641		80,357	
Alveolar bone	27,052		49,982	
Aligner	20,566	21,057	40,786	41,519
Total	130,164	130,655	234,992	235,725

With regards to boundary conditions and restrictions, the movement of maxilla bone was restricted for all degrees of freedom at its superior region.

With regards to contact conditions, a rigid union condition without relative displacement (bonded) was established at the following interfaces: ligament-bone, tooth-ligament, and teeth-attachments. A ‘no separation’ condition was constructed among these different interfaces. A frictionless condition was established at the contact interfaces between the aligner and the tooth crown surface and its attachments.

### Material properties related to modeling

For simulation of the involved structures, we determined the physical characteristics in the following manner (Table 2). Alveolar bone: A linear elastic isotropic and homogeneous behavior was assumed, and mechanical properties were obtained from previous studies [24, 25]. Differences in rigidity between different bone types were not contemplated as these were not considered relevant to the objectives of the study. Teeth (the maxillary dentition with extracted first premolars): A linear elastic isotropic and homogeneous behavior was assumed, and mechanical properties were obtained from previous studies [24, 25]. Differences in rigidity between the enamel, dentin and cement were not contemplated as these were not considered relevant to the objectives of the study. PDL: We assumed an isotropic homogeneous linear elastic behavior. Mechanical properties were inferred from previous studies [24, 25]. It is worth noting that most researchers still agree that it is reasonable to study tooth movement tendency even if the PDL is assigned linear material properties in FEA [12, 26, 27]. Clear aligner and composite attachments: We assumed a linear elastic isotropic and homogeneous behavior. Mechanical properties were inferred from previous studies [15–17, 24, 25].

**Table 2:** Properties of the materials considered in this study

Material	Young’s Modulus, MPa	Poisson Ratio
Alveolar bone	1.37 × 10^3^	0.3
Tooth	1.96 × 10^4^	0.3
PDL	6.9 × 10^−1^	0.45
Aligner	816	0.36
Attachment	12.5 × 10^3^	0.36

### Establishment of a coordinate system

The X-axis represented the direction of the coronal plane with the positive direction being towards the mesial surface of the tooth; the Y-axis represents the sagittal plane with the positive direction being towards the lingual surface, and the Z-axis represents the vertical plane with the positive direction being towards the gingival tissue.

### Configuration settings

Two groups of models were created: (1) for the 0.5 mm-thick aligner and (2) for the 0.75 mm-thick aligner. The maxillary crown and attachment were extended outwards by 0.5 mm and 0.75 mm to simulate the aligners.

### Loading method

In this FE simulation of a clinical event, a retraction of 0.25 mm in the sagittal direction was imposed on the initial anterior region; this drove the deformation of the aligner segment. The forces generated by the aligner on each tooth were then calculated by software (ANSYS, Pennsylvania, USA) and were then loaded back on the corresponding tooth in the reverse direction.

### Experimental design

Two group sets were designed in this study. Vertical rectangular attachments were designed on the buccal surfaces of canines, second premolars, first molars and second molars for all models generated in this study. For the first group set, the aligner thickness was set as 0.5 mm; a group without a power ridge was set as a control group. Six groups with different power ridges height (power ridges own thickness) (0.3 mm, 0.5 mm, 0.6 mm, 0.7 mm, 0.8 mm and 1.0 mm) were also used as carriers to mimic torque control. The central incisors were the main object of observation in this study. Therefore, the power ridge was set on the labial surface of the central incisors. The location and dimension of the power ridge are shown in Fig. 1. The rotation angle in the sagittal direction of the central incisor was observed. When this rotation angle was 0°, the displacement trend was translation and the corresponding power ridge height was selected. For the second group set, the grouping details were similar to the first group set, except that the aligner thickness was set as 0.75 mm and the height of the power ridges was 0.1 mm, 0.2 mm, 0.25 mm, 0.3 mm, 0.4 mm and 0.5 mm, respectively. When this rotation angle was 0°, the displacement trend of the central incisor was translation, and the corresponding power ridge height was selected. The influence of aligner thickness on the height of power ridges (the magnitude of torque compensation) of the central incisor with translation was recorded, and the displacement tendency and the principal stress of the incisors, canines and second premolars were observed. The incisal and apical center of the anterior teeth and the occlusal center and apical center of the buccal root of the second premolars were taken as the measuring points.

## Results

**Fig. 2:**
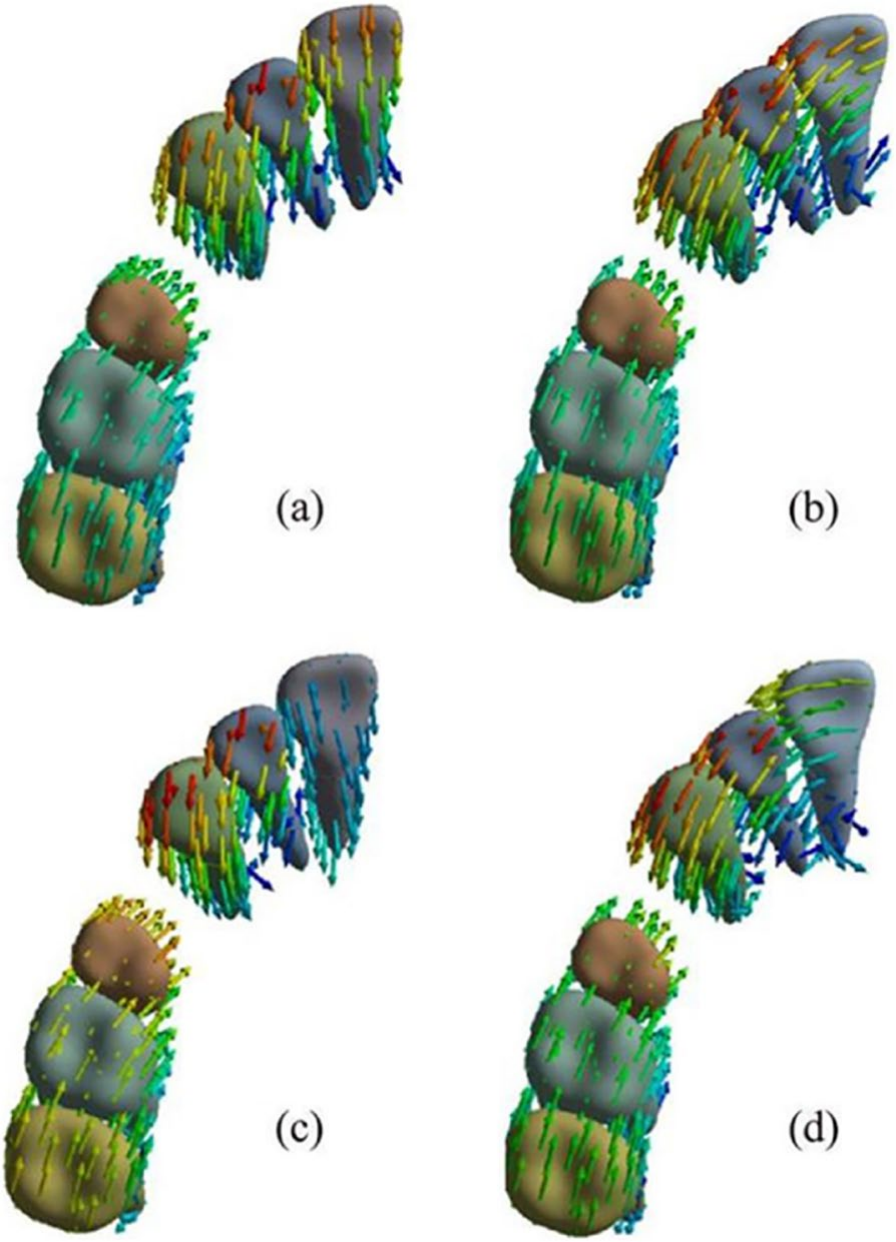
the displacement tendency of the maxillary dentition. **a** without torque compensation-0.5 mm-thick aligner; **b** without torque compensation − 0.75 mm-thick aligner; **c** with torque compensation-0.5 mm-thick aligner; **d** with torque compensation-0.75 mm-thick aligner. Arrows indicate the direction of tooth movement；the colder the tone, the less the tendency for tooth movement; the warmer the tone, the less the tendency for tooth movement. The 0.75 mm-thick aligner produced more significant distal tipping tendency for central incisors

**Fig. 3:**
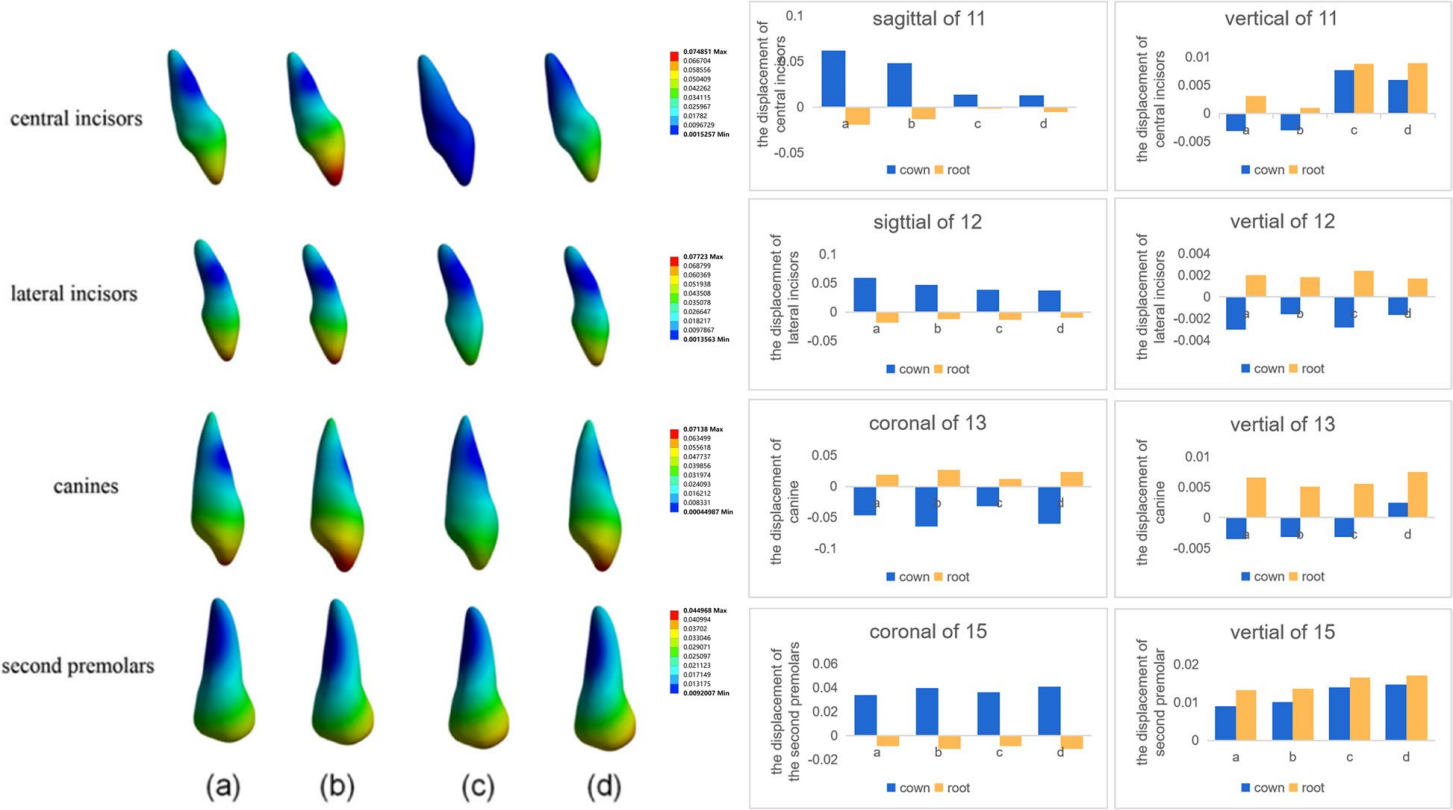
Displacement tendencies of teeth with and without torque compensation under the condition of different-thick aligners. **a** The displacement tendency of teeth with the 0.5 mm-thick aligner before torque compensation. **b** The displacement tendency of teeth with the 0.75 mm-thick aligner before torque compensation. **c** The displacement tendency of teeth with the 0.5 mm-thick aligner after torque compensation. **d** The displacement tendency of teeth with the 0.75 mm-thick aligner after torque compensation

When there was no torque compensation, the incisors exhibited distal tipping, lingual tipping, and extrusion; canines were distally tipped and extruded; and second premolars were mesially tipped and intruded (Fig. 2(a)(b)). The rotation angle in the sagittal direction for central incisors with the 0.75 mm-thick aligner was 0.1262°; this was smaller than that with the 0.5 mm-thick aligner (0.1688°) (Table 3). In other words, the magnitude of torque loss with the 0.75 mm-thick aligner was smaller than that with the 0.5 mm-thick aligner for the central incisors. Moreover, the displacement of the central incisors in a sagittal direction with the 0.5 mm-thick aligner (0.0618 mm) was larger than that with the 0.75 mm-thick aligner (0.0482 mm) (Fig. 3). A clear aligner also produced distal tipping for central incisors; this tendency was more significant with the 0.75 mm-thick aligner than that with the 0.5 mm-thick aligner (Fig. 2(a)(b)). Furthermore, extrusion of the central incisors occurred due to the tipping of these teeth; this was more significant with the 0.5 mm-thick aligner (Fig. 3). Similar to the central incisor, the lateral incisor also exhibited retraction, distal tipping and extrusion; these effects were less significant when compared with the central incisor. The effects on the canines when distally tipped were more significant with the 0.75 mm-thick aligner，while the effects on the canines when extruded were more significant with the 0.5 mm-thick aligner. A clear aligner produced mesially tipping and intrusion for second premolars; this was more significant with the 0.75 mm-thick aligner (Fig. 3).

**Table 3:** The influence of aligner thickness on the torque movement of central incisors

Torque compensation	Without		With	
Aligner thickness (mm)	0.5	0.75	0.5	0.75
The rotation angle of central incisors in sagittal direction (°)	−0.1688	− 0.1262	0	0
The corresponding power ridge height (mm)	0	0	0.7	0.25

**Fig. 4:**
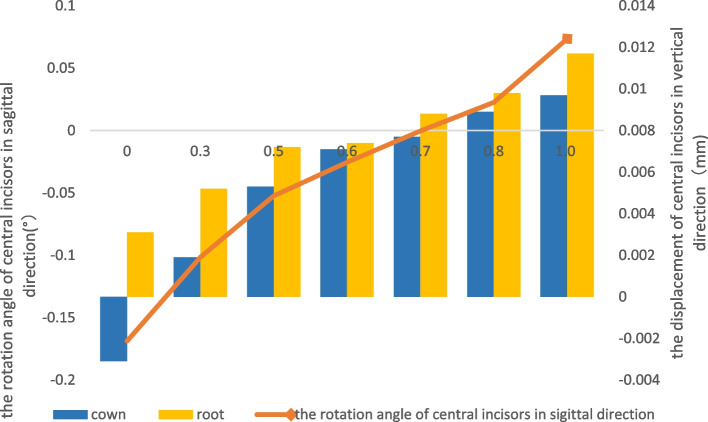
The displacement tendency in sagittal and vertical direction for central incisors with different height power ridges by 0.5 mm-thick aligner. The right ordinate represented the rotation angle of central incisors in sagittal direction (°), the left ordinate represents the displacement of central incisors in vertical direction(mm), and the abscissa represents the height of the power ridge(mm); The power ridge was 0.7 mm when bodily movement was achieved for central incisors with 0.5 mm-thick aligner

**Fig. 5:**
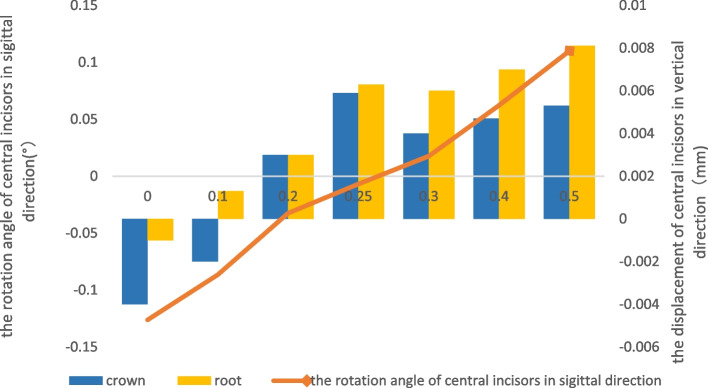
The displacement tendency in sagittal and vertical direction for central incisors with different height power ridges by 0.75 mm-thick aligner. The right ordinate represented the rotation angle of central incisors in sagittal direction (°), the left ordinate represents the displacement of central incisors in vertical direction(mm), and the abscissa represents the height of the power ridge(mm); The power ridge was 0.25 mm when bodily movement was achieved for central incisors with 0.75 mm-thick aligner

When the power ridge height was 0.7 mm, the displacement tendency of the central incisors with the 0.5 mm-thick aligner was translation (Table 3) (Fig. 4). A power ridge height of 0.25 mm supported bodily retraction for the central incisors with the 0.75 mm-thick aligner (Table 3) (Fig. 5). This meant that the thicker aligner generated less torque loss and required less torque compensation; the thicker aligner magnified the effect of selective modifications, such as power ridges on the cervical area. Significantly, the displacement tendency of intrusion for the central incisors increased as the power ridge height increased (Figs. 4 and 5). Furthermore, torque compensation produced less distal tipping and more intrusion for the canine and the magnitude of the reduction in distal tipping and the increase in intrusion was more significant with 0.75 mm-thick aligner. However, power ridges had less influence on the lateral incisors (Fig. 3). The mesial tipping and intrusion of the second premolars was positive as the thickness of the aligner and the magnitude of torque compensation increased (Fig. 3).

**Fig. 6:**
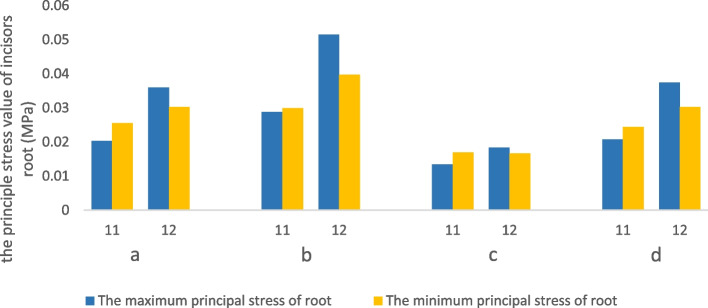
The influence of aligner thickness on the stress value of PDL for incisor. **a** The principal stress of the anterior teeth with the 0.5 mm-thick aligner under the condition of no torque compensation. **b** The principal stress of the anterior teeth with the 0.75 mm-thick aligner under the condition of no torque compensation. **c** The principal stress of the anterior teeth with the 0.5 mm-thick aligner under the condition of torque compensation. **d** The principal stress of the anterior teeth with the 0.75 mm-thick aligner under the condition of torque compensation

**Fig. 7:**
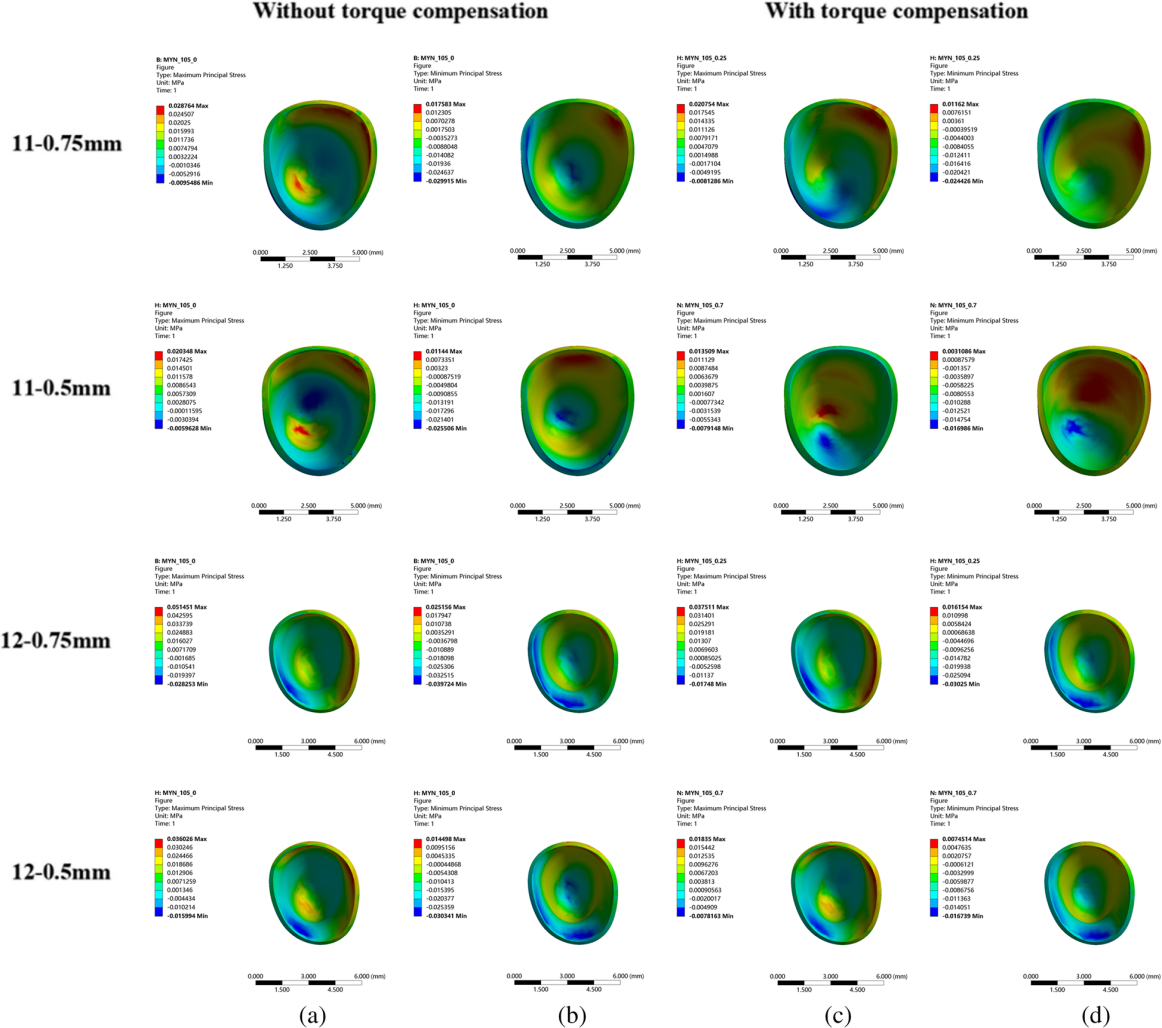
The influence of aligner thickness on the stress distribution of the PDL for incisors. **a** the maximum principle stress of incisors without torque compensation. **b** the minimum principle stress of incisors without torque compensation. **c** the maximum principle stress of incisors with torque compensation. **d** the minimum principle stress of incisors with torque compensation

For incisors, with no aligner torque compensation, the maximum principal stress was mainly generated on the apical area of palatal surfaces and the cervical area of the labial surface. Clear aligners mainly produced the minimum principal stress on the apical area of the labial surface and cervical area of the palatal surface. In addition, the magnitude of PDL stress was larger with a 0.75 mm-thick aligner than that with the 0.5 mm-thick aligner (Fig. 6). However, there was no significant difference with regards to the distribution of PDL stress between the 0.75 mm-thick aligner and the 0.5 mm-thick aligner (Fig. 7). Under aligner torque compensation, the 0.75 mm-thick aligner resulted in a more evenly distributed stress for incisors than that with the 0.5 mm-thick aligner (Fig. 7). The maximum principal stress and the minimum principal stress of the PDL for incisors all decreased when compared with the case without torque compensation; the stress induced by the 0.75 mm-thick aligner was higher than that with the 0.5 mm-thick aligner (Fig. 6).

## Discussion

The objective of this study was to identify the effects of torque compensation on the incisors with different thicknesses of clear aligner on the three-dimensional displacement tendency of teeth in cases of extraction. A previous clinical study proved that there was a higher incidence of “roller-coaster effect” in extracted cases with clear aligners which was associated with the lingual tipping and extrusion of incisors, the distal tipping of canines and the mesial tipping of the posterior teeth [16, 28]. These previous findings were consistent with the present study in which we found that clear aligners produced retraction, lingual tipping, distal tipping and extrusion of the incisors, distal tipping and extrusion of the canines, and mesial tipping and intrusion of the second premolars. In a previous study, Lai explored the occurrence mechanism of “roller-coaster effect” with clear aligners [16] and found that these aligners produced a retraction force (on anterior teeth) and a protraction force (on posterior teeth) that were applied on crowns and passed through the occlusal side of the center of resistance, thus resulting in “roller-coaster effect” .

Furthermore, as shown in Fig. 3, we detected a significant difference in the tendency of incisors displacement in the three-dimensional direction between the 0.75 mm-thick aligner and the 0.5 mm-thick aligner before torque compensation. First, the 0.75 mm-thick aligner produced more significant distal tipping tendency. A previous study demonstrated that thicker aligners produced stronger orthodontic forces under the same magnitude of deformation [20]. Logically, the orthodontic force in the distal direction produced by 0.75 mm-thick aligners for incisors was more significant. Second, the magnitude of torque loss with the 0.75 mm-thick aligner was smaller than that with the 0.5 mm-thick aligner while the magnitude of sagittal displacement with the 0.75 mm-thick aligner was also smaller than that with the 0.5 mm-thick aligner; this could be explained by the fact that the 0.75 mm-thick aligner generated a stronger control force in the sagittal direction. In addition, the 0.5 mm-thick aligner and the 0.75 mm-thick aligner produced extrusion of the incisors. In the clinical scenario, an air gap between an aligner and the dentition is filled with saliva, thus causing friction between the aligner and the dentition. The friction generated by the diminished fit of the appliance could be used to explain the extrusion of teeth. Although the models were established with the same Young’s modulus values, the rigidity of the aligner increased as the thickness of the aligner increased [29]. Therefore, the thicker aligner produced less deformation and more intrusion force; this which correlated with the fact that the extrusion tendency of the incisors was less significant with a 0.75 mm-thick aligner than with the 0.5 mm-thick aligner. Although this study did not take friction into consideration, the extrusion tendency of the incisors with aligners of different thicknesses was of significant note for orthodontic clinical practice (Fig. 4).

Bodily retraction of relative upright incisors was also frequently encountered among extraction cases; this is a complicated tooth movement that requires adequate intrusion and torque control of the incisors [30]. Simon et al. reported that root torque and the intrusion of incisors could not be fully achieved by clear aligners (42%) [31]; the Vincenzo’ sample torque for the central incisors and canines accounted for just 35% of movements [32]. These results showed that additional torque control of the incisors should be designed to increase the predictability of tooth movement. From the perspectives of biomechanics, Hahn et al. considered that controlling the torque of an upper central incisor requires the creation of effective couples: a tipping force was required, evoked by reversible deformation of the appliance near the gingival margin and the resulting force in the opposite direction produced by movement of the tooth against the inner opposite surface of the appliance near the incisor edge [33]. However, the force couple generated at the cervical and incisal regions of the aligners is not large enough to generate an adequate counter moment due to limitations imposed by material properties [17, 34]. This situation could eventually be improved by selective modifications to aligners by means of mechanical reinforcement of the cervical area [34], such as the power ridge of the Invisalign system. A previous study found that the power ridge generated significant torque compensation (7.9 N.mm) during torque movement of the upper central incisors, thus confirming the efficiency of the power ridge for anterior teeth torque [35].

In the present study, we proved that the central incisors exhibited bodily retraction when the power ridge height was 0.7 mm with the 0.5 mm-thick aligner and 0.25 mm with the 0.75 mm-thick aligner. In essence, the presence of a power ridge in the tooth neck produced a counter moment because it generated an angle between the inner face of the aligner and the tooth surface; this led to further deformation of the aligner. From mechanical knowledge, this angle was determined by the height of the power ridge and reflected the magnitude of torque control [17]. This indicated that the 0.75 mm-thick aligner generated less torque loss and required less torque compensation, that is, the thicker aligner magnified the effect of the selective modification. In the vertical direction, the central incisors exhibited absolute intrusion. The magnitude of intrusion for the central incisors increased as the height of the power ridge increased (the magnitude of torque compensation). Lateral incisors exhibited relative extrusion with both the 0.5 mm-thick aligner and the 0.75 mm-thick aligner. In other words, the torque compensation was ineffective on the lateral incisors; this could be attributed to the off-tracking effect of the clear aligners in that the shape of the clear aligner with torque compensation did not match the lateral incisors [16]. Compared with the 0.5 mm-thick aligner, the 0.75 mm-thick aligner produced more distal tipping and intrusion for the canines after torque compensation; this was consistent with the central incisors. This was mainly due to the fact that the rectangular attachment on the canines guaranteed a sufficient contact area between the canines and the aligner. What’s more, on one hand, compared with no torque compensation, power ridges made the aligner further distorted, thus generating stronger orthodontic forces. On the other hand, a power ridge of 0.25 mm produced less “off-tracking effect” of the clear aligners than 0.7 mm power ridges. Therefore, the 0.75 mm-thick aligner with torque compensation produced more mesial tipping and intrusion for the second premolar. Consequently, the 0.75 mm-thick aligner was more suitable if the incisors were relatively upright in cases of extraction, and more attention should be paid to retentive force at the lateral incisor and the anchorage control considering the more significant mesial tipping tendency of the second premolar.

A previous study highlighted that the incidence of root resorption (RP) after orthodontic treatment was 91% [36]. Furthermore, the intrusion and root torque control of the incisors is known to increase the incidence of RP [16, 37]. It has also been reported that maxillary incisors are more susceptible to root resorption [16]. Therefore, more attention should be paid to the RP of incisors in cases of extraction with relative upright incisors. The force stress that is concentrated on the root surfaces is known to be the main culprit for root resorption [38]. The hydrostatic stress of the element in PDL could be used to predict the area where RP may occur [36]. This was confirmed by Wu et al. who pointed out that the cell pressure caused by the optimal orthodontic loading in PDL should not be higher than the capillary pressure in tissue [39]. Generally, cell pressure and capillary pressure requires principal stress instead of Von-mises stress [40–42]. Based on these factors, the present study calculated and compared the maximal principal stress of the PDL to analyze RP of the incisors. We found that the stress of the incisors was concentrated on the apical and cervical areas without torque compensation; this was the typical stress distribution for lingual tipping movement. With torque compensation, the magnitude of PDL stress decreased; this could be explained by the fact that appropriate aligner torque compensation resulted in the bodily retraction of the incisors and the retraction force was applied on the whole palatal root surfaces. This phenomenon was consistent with the conclusion reported by Lai [16]. Significantly, the 0.75 mm-thick aligner with torque compensation produced a more even distribution for the incisors. This indicated that appropriate torque compensation with a thicker aligner reduced the incidence of resorption.

## Limitations

This study innovatively investigated the influence of aligner thickness on the torque control and intrusion of incisors with clear aligners and provided valuable clinical guidance relating to the “roller-coaster effect” in cases of extraction from the perspective of aligner thickness. However, our findings must be clinically validated to support the use of new types of plastic aligners with appropriate evidence. One of the limitations was that the present study did not consider certain biological questions, such as the long-term application of orthodontic forces，the root length, root morphology and the specific properties of the material used to make the aligner. Furthermore, the evaluation of only one model, as well as two types of aligner thickness, could be considered as another limitation of the present study. In addition, an air gap between an aligner and the dentition is filled with saliva; the presence of saliva influenced the friction between the aligner and the dentition. The air gap and saliva were not considered in the current model. Therefore, future research should consider these issues.

## Conclusion

Clear aligner therapy produced the “roller-coaster effect” during anterior retraction in cases of extraction. Torque compensation caused by power ridges can achieve incisor intrusion and palatal root torque. In cases of extraction, appropriate torque compensation with a thicker aligner should be designed to ensure bodily retraction of anterior teeth and minimize root resorption, but more attention should be paid to the anchorage control of the posterior teeth.

## Data Availability

The datasets used and/or analyzed during the current study are available from the corresponding author on reasonable request.

## Abbreviations

FEA: Finite Element Analysis
PDL: Periodontal ligament
CAT: Clear aligner therapy
CAD: Computer aided design
CBCT: Cone Beam Computed Tomography
RP: Root resorption
